# A survey of skin tone assessment in prospective research

**DOI:** 10.1038/s41746-024-01176-8

**Published:** 2024-07-17

**Authors:** Vanessa R. Weir, Katelyn Dempsey, Judy Wawira Gichoya, Veronica Rotemberg, An-Kwok Ian Wong

**Affiliations:** 1 Dermatology Service, Memorial Sloan Kettering Cancer Center, New York, NY USA; 2 Department of Medicine, Division of Pulmonary, Allergy, and Critical Care Medicine, Duke University, Durham, NC USA; 3 Department of Radiology and Imaging Sciences, Emory University School of Medicine, Atlanta, GA USA; 4 Department of Biostatistics and Bioinformatics, Division of Translational Biomedical Informatics, Duke University, Durham, NC USA

**Keywords:** Medical research, Clinical trial design, Translational research

## Abstract

Increasing evidence supports reduced accuracy of noninvasive assessment tools, such as pulse oximetry, temperature probes, and AI skin diagnosis benchmarks, in patients with darker skin tones. The FDA is exploring potential strategies for device regulation to improve performance across diverse skin tones by including skin tone criteria. However, there is no consensus about how prospective studies should perform skin tone assessment in order to take this bias into account. There are several tools available to conduct skin tone assessments including administered visual scales (e.g., Fitzpatrick Skin Type, Pantone, Monk Skin Tone) and color measurement tools (e.g., reflectance colorimeters, reflectance spectrophotometers, cameras), although none are consistently used or validated across multiple medical domains. Accurate and consistent skin tone measurement depends on many factors including standardized environments, lighting, body parts assessed, patient conditions, and choice of skin tone assessment tool(s). As race and ethnicity are inadequate proxies for skin tone, these considerations can be helpful in standardizing the effect of skin tone on studies such as AI dermatology diagnoses, pulse oximetry, and temporal thermometers. Skin tone bias in medical devices is likely due to systemic factors that lead to inadequate validation across diverse skin tones. There is an opportunity for researchers to use skin tone assessment methods with standardized considerations in prospective studies of noninvasive tools that may be affected by skin tone. We propose considerations that researchers must take in order to improve device robustness to skin tone bias.

## Introduction

Technological advances have made non-invasive medical devices (e.g., pulse oximetry, heart rate monitors, artificial intelligence-based diagnostics) irreplaceable aspects of modern patient care. However, evidence shows many of these devices are susceptible to skin tone bias, which can worsen disparities in outcomes^1^. Modalities relying on transcutaneous measurements may produce bias due to skin tone variation and device validation on non-diverse populations, limiting device performance and overall generalizability^1,2^.

Recent evidence shows current racial bias in pulse oximetry. A retrospective analysis revealed a 3x increased frequency of undetected hypoxemia in Black patients (17%; 95% CI 12.2–23.3) compared to white patients (6.2%; 95% CI 5.4–7.1)^3^. Although there are mixed results^4,5^, several studies show overestimation in arterial oxygen saturation by 0.17–10% in darkly pigmented subjects, especially at lower SpO_2_ values^6–8^. Furthermore, darker skin tone is associated with a larger bias of undetected hypoxemia^9^.

Artificial Intelligence (AI) image classification is an emerging non-invasive tool that aims to improve diagnostic accuracy in medicine^10^, including classification of skin lesions^10–12^. However, increasing literature recognizes bias in their performance, with models performing worse on individuals with darker skin tones^13^. Daneshjou et al. showed that three state of the art algorithms had performance decreases on darker skin types (FST V, VI) compared to lighter skin types (FST I, II) (ModelDerm: 0.55 vs 0.64; DeepDerm: 0.50 vs 0.61; HAM10000: 0.57 vs 0.72)^14^. Groh et al. further exemplified that models are most accurate for skin types they were trained on, although some studies report no differences in model performance by skin tone^15^. The effect of skin tone on model performance is largely underreported—only 10% (7/70) of deep learning algorithms include information about skin tone^16^ and few report performance by skin tone categories^17^. Further, there is no gold standard for skin tone labeling, and commonly used practices like estimated Fitzpatrick Skin Type are limited by uncertainty^18^ and lack of inclusiveness. (ref. ^11^; ref. ^12^; ref. ^10^; ref. ^19^)

To improve generalizability of assessments and algorithm fairness, it is critical that patient skin tone variation be taken into account in validation studies of noninvasive technologies. There are initiatives to modify device monitoring regulation criteria, such as those released by the FDA in November 2023^20^. In this review, we will (1) briefly present a review of background and methods for skin tone measurement within health care then (2) provide detailed study considerations for measuring skin tone in prospective trials.

## Results

### Part I. Review of skin tone assessment

#### Defining skin tone

Color is the perception of light based characterizations, such as hue, lightness, and saturation. Physiologically, the inherent color of the skin, defined as “skin tone”, is the result of light absorbing compounds called chromophores. The most abundant chromophores in humans are melanin (pheomelanin and eumelanin), carotene, oxygenated hemoglobin, and reduced hemoglobin^21^. In general, the two major contributors to skin tone are melanin, which produces a brown tint, and hemoglobin, which creates red and purple-blue hues^22^. Frequent methods for discriminating skin tone for the purpose of validating noninvasive assessment tools are administered visual scales and color measurement tools^23^ (Table 1). Skin tone can also be extracted from camera images through a variety of techniques^24^. For the purposes of this paper, automated skin color extraction, modeling, and labeling will not be discussed^23,25,26^.

#### Administered visual scales

Administered visual scales, such as Von Luschan, Monk, and Pantone, utilize numbered colored tiles that are matched to a person’s skin tone (Table 1). Fitzpatrick Skin Type (FST) was originally developed to assess tanning and burning propensity, however, many use FST as a proxy for skin tone^27^ despite evidence showing that FST is poorly correlated with objective measurements of skin color evaluation^27,28^. Although widely available and inexpensive to administer, visual scales can be limited by subjectivity. Furthermore, visual scales can be affected by complex human perception of color which is influenced by light, anatomic site, the context of the object, or a person’s unique experiences with similar objects^29–32^.

**Table 1:** Descriptions of common skin tone measurement tools. This is a nonexclusive list

Scale	Description	Applications	Pros	Cons	Citations
Administered					
Fitzpatrick skin type Scale	Classifies skin on a six point scale [I (always burns, never tans) to VI (never burns, tans deeply)] and assumes a reciprocal relationship between burning and the tendency to tan	- Assessment of tanning and burning propensity in response to UV therapy -Assess skin cancer risk, and predict tolerance to therapies (laser, peels) - Incorrectly used as proxy for skin color	-Available online and free -Widely used	- Incorrectly used as proxy for skin color -Originally designed only for non-hispanic whites (V, VI added later)	^63–65^
Von Luschan Chromatic Scale	Color scale 1 (very light) to 36 (very dark)	- Originally invented for anthropometry studies.	- Available online and free to use	- Previously used to support scientific racism - Requires color accurate printing	^66,67^
Monk Scale ®	The 10-shade open source scale was created by sociologist Ellis Monk with Google AI	- Created for image-based or in person skin tone labeling	- Available online, free to use, and shows high reliability - Used similarly between experts and an average person	- Shade selection varies systematically based on geographic region - Requires color accurate printing - Limited shade range	^55,68,69^
Pantone Scale ®	Handheld visual skin color guide of 138 unique colors which vary based on chroma (1–5), hue (Y/R), and lightness (1–15)	- Used within the cosmetic industry, for prosthetics matching, and in computer graphics.	- Large shade range - Defined and standardized color space	- Must be purchased - Size may affect reliability and increase measurement time	^54,70,71^
Color measurements					
Colorimeter					
Konica Minolta Chromameter CR-400 ®	- Handheld colorimeter designed to measure smooth surfaces with low color variation - Measurement surface: 8 mm - Provides readings in XYZ, YXY, L*a*b, Hunter lab, L*C,h, Munsell, CMC, CIE1994, Lab99, LCh99, CIE2000, CIE WI-Tw, WI ASTM E313, YI ASTM D1925, YI ASTM 313	- Extensively used in dermatologic applications for color evaluation - Other applications include food, building materials, and plastic quality control	- Portable, handheld, lightweight - Six language options - Integrated software to record measurements	- Larger than other colorimeters (~540 g) - Needs further validation on skin tone	^72^
Delfin SkinColorCatch ®	- Colorimeter made for skin color evaluation - Colorimeter that utilizes white LEDs at a 45 degree angle to capture reflected light with an RGB color sensor. - Measurement area: 0.3 cm^2^ - Provides ITA, erythema index (EI), melanin index (MI), CIEL*a*b*, chroma, hue, and RGB	- Used to evaluate skin tone, pigmentation, burn and scar assessment, and to establish a ground truth to train automatic Fitzpatrick labeling	- Designed specifically for skin tone assessment - Calculates ITA automatically - In-vivo and in-vitro measurements - Integrated software to record measurements	- Need further validation in relation to skin tone	^73^
Antera 3D®	- Camera that uses multidirectional illumination and spectral reflectance mapping of 7 wavelengths to derive chromophore concentrations and colorimetric data for skin - Measurement area: 56 mm × 56 mm - Provides an erythema parameter, melanin parameter, and colorimetric data in CIEL*a*b*	- Used in evaluation of skin aging, dyschromia response to lasers/topical agents, keloid treatment	- Provides simultaneous photos and colorimetric data - Can also quantify skin type wrinkles, texture, scars - Can measure changes in parameters over time	- Need further validation in relation to skin tone	^74,75^
Courage-Khazaka Skin Colorimeter Flex Probe CL-440®	- Colorimeter made for skin color evaluation - Measurement surface: 7 mm and 13 mm - Provides values in CIEL*a*b* and ITA	- Used to evaluate skin tone and in topical treatment efficacy evaluations for melasma, hyperpigmentation, and burns	- Device has interchangeable tips -Light-based “placement aid” to specify exact measurement area - Automatic calculation of ITA - Integrated software to record measurements	- Need further validation in relation to skin tone	^76^
Spectrophotometer					
Konica Minolta CM-700d®	- Spectrophotometer made to sample color and appearance on varying surface types - Measurement surface: 8 mm and 3 mm Light sources: A, C, D50, D65, F2, F7, F8, F10, F11, F12 - Provides spectral curves, L*a*b, L*C*h, Hunter Lab, XYZ, Munsell, MI, WI, YI, ISO Brightness, and 8 gloss value	- Used for quality control, color analysis, process control in both manufacturing and research, including dermatologic applications	- Option for ISO 17025 calibration - Easily operated - Handheld, portable device - Device has interchangeable tips - Compatible with measurement recording software, and CM-SA skin analysis software	- High cost tool - Need further validation in relation to skin tone	^77^
Variable Spectro®	- Compact low-cost spectrophotometer using multiple light sources to capture color details - Provides spectral curves, CIEL*a*b* values, and color matches to libraries including Sherwin-Williams, Behr, and PPG - Measurement surface: 8 mm - Four light sources : A, F2, D50, and D65	- Used for patching paint colors, food, and material quality control	- Cheaper than other color measurement devices - Easily operated - Handheld, portable device - Record measurements with phone application	- Not commonly used in skin tone measurement; needs further validation in relation to skin tone	^78^
Mexameter MX 18®	- Narrow band spectrophotometer that measures reflection from 3 emitted wavelengths Measurement surface: 5 mm ≈ 19.6 mm² -Provides erythema index (EI) and melanin index (MI)	- Widely used within dermatology including to measure skin tone, hyperpigmentation, and rosacea	- Can perform continuous measurements - Easily operated - Handheld, portable device - Spring in measurement head maintains low contact pressure	- Need further validation in relation to skin tone	^79^
Cameras					
Commercial Digital					
Smartphone (iPhone, Android)	High end phone with multiple rear facing cameras	- Widespread	- Widely available - Often multiple cameras - Easy to export	- Color not consistent across models - Potential disclosure of PHI	^80^
Basic or Advanced Compact camera	Simple to use camera that offers automatic or manual modes	- Widespread	- Relatively inexpensive - Portable - Easy to use - Some manual control	- Decrease image quality compared to advanced cameras - Fixed lens	^80^
Digital single lens reflex (DSLR)	Digital single lens reflex camera made with interchangeable lenses capable of capturing professional quality photos	- Widespread	- Full manual control - Can export in RAW	- Expensive - Can be difficult to use	^80^
Specialist					
Dermatoscope	Common device used in dermatology that employs light and magnification to evaluate skin lesions, hair, and nails	- Diagnosis of pathology of the skin and nails	- Shows details in the outer layer of skin that would not be visible to the naked eye	- Expensive	^81^
3D Total Body Photography	3D total body photography that captures most of the body’s surface in macro-quality resolution	- Mapping and monitoring lesions and skin conditions over time	- Fast capture of entire body - Preliminary software that offers skin analysis and lesion tracking over time - Polarized and non-polarized lighting to help reduce reflection	- Expensive	^82,83^

#### Color measurement tools

Color measurements are objective measurements achieved through reflectance spectrophotometry (Konica Minolta CM700D, Variable Spectro) and colorimetry (Delfin SkinColorCatch) (Table 1). In current works, color measurement tools are being utilized as a by-product of the limitations of visual scales by to providing objective measurements to increase precision in color quantification^33–35^. Color measurements offer greater color precision, but the tools are expensive and devices are sensitive to environmental influences^25,26^.

#### Cameras and color spaces

Several differing color models provide a framework to systematically or mathematically describe color output. One of the most common, the RGB model (red, green, blue), was developed to mimic the primary colors perceived by the eye^32^. It encodes color in an additive fashion where a combination of all three colors results in white. Other color models include HSL (hue, saturation, lightness), CIELAB (lightness, green-red gradient, blue-yellow gradient), and CIECAM02 (brightness, lightness, chroma, saturation, hue, and “colorfulness”)^32,36^. Most reproduction of color on printed work is exported in a CMYK (cyan, magenta, yellow, black) color space. Color spaces are a specific organization of colors that are mapped to values in color models in a standardized way. The standardized RGB color space (sRGB) is the most commonly used space for representing digital images on displays^26,37^.

### Part II. Considerations for study design

#### Body part assessed

A summary of recommendations for skin tone measurement can be found in Table 2. Unaltered skin tone represents a combination of genetic factors and environmental influence based on constitutive (baseline skin color) and facultative (skin color altered by sun exposure) grouping. Constitutive skin color is best characterized in sun-protected areas more likely to represent unaltered baseline pigmentation^38^. However, one’s perceived skin tone may also be influenced by exogenous factors including artificial tanner, makeup, or tattoo pigmentation. Depending on the technology being validated, the inclusion of at least one constitutive skin site may be important given its decreased variability across seasons and increased correlation with skin phototype^39^. The upper volar arm has been proposed as a reliable measurement of constitutive skin given its low seasonal variability and ease in access^40^. Otherwise, body part selection may be predetermined based on the application (e.g., using the finger/earlobe in pulse oximetry).

**Table 2:** Considerations for skin tone measurement in prospective research studies

Considerations for a Prospective Study	Conclusions
Patient Factors	
Body Part Assessed	- Include sun-exposed and non sun-exposed body sites for skin tone measurement - Assess bruising and scarring - Survey tanning ability
Underlying Conditions	- Document or exclude patients with pigmentary disorders or medical conditions affecting chromophores (jaundice, severe anemia) depending on outcome of interest
Perfusion-related Color Changes	- Avoid pressure when using skin tone assessment tools - Allow patients to be immobilized to equilibrate to the environment
Considerations for Study Design	
Clinical Location	- Outpatient settings may have more mobile patients and standardized environments - Inpatient setting may have less standard environments, less mobile patients, and less accessible body parts for skin tone measurement
Ambient Lighting	- Create a standardized lighting environment. - Artificial lighting with similar temperature to natural light (5000-6500 K) is suggested
Timing of Measurements and Outcomes	- Data should be time synchronization for skin tone measurement with other collected variables.
Longitudinal Measurements	- For longitudinal study designs, patients should avoid sun exposure. - Standardize environment and tools across timepoints for consistent color evaluation
Dataset Balancing	- Race should not be used as a proxy for skin tone - Datasets should be balanced by skin tone and race to account for disparities from either factor
Considerations for Measurement Tools	
Choice of Administered Scale	- Administered scales are low cost, widely available, are useful in prospective and post-hoc analysis, but can be influenced by user perception - Choice of administered scale will depend on price, size, range, and tool consistency - Larger scales may have more granularity but decreased inter-rater reliability
Choice of Color Measurements	- Colorimetric and spectrophotometric devices are easily-operated and non-invasive - Color space measurements can be interconverted allowing for data sharing - Several colorimeters and spectrophotometers exist and choice may depend on device size, cost, and clinical setting - Able to quantify erythema, melanin, pigmentation, and skin color
Choice of Camera	- Camera choice of commercial (iPhone, DSLR) vs specialized (dermatoscope, VECTRA, VISTA) may depend on price, access, and setting - Camera settings, including aperture, shutter speed, lighting, and image format should be standardized - White balance is a critical modifiable factor, thus one may consider using color calibration cards in the image frame
Analysis	
Image Processing	- JPEG image storage may increase computational efficiency but can lead to compression artifact - RAW storage can help maintain color consistency, but is may not be feasible for capture
Tool Reliability	- Use triplicate measurements for color measurements - Use at least two raters for administered visual scales
Data Transparency	- Consider publishing de-identified skin tone meta-data for open data reuse

#### Underlying conditions that can affect skin tone

Several conditions can affect the relative concentration and distribution of chromophores and alter skin tone assessment. Therefore, study designs incorporating skin tone measurement should consider pigmentary disorders (e.g., vitiligo or melasma) and medical conditions (e.g., anemia and jaundice) that influence skin tone. Perfusion-related changes in skin (e.g., flushing, blanching) can also affect skin tone assessment^34^. To minimize these effects, it is recommended that skin measurements occur in a pressure-free state and at rest, and to collect as much information about factors that influence skin tone at the time of measurement as feasible.

#### Ambient lighting

The impact of lighting on the perception of color is critical and commonly overlooked in study design. Ambient lighting can come in various forms, such as brightness and temperature. Ambient lighting can influence color perception based on time of day and location and may skew skin tone perception, making it appear lighter or darker^41^. Ambient lighting should be both sufficient and standardized to increase the accuracy and precision of skin tone assessment. To prevent variability in daylight conditions, artificial lighting with similar temperature to natural light (5000–6500 K) could be helpful^42^. A controlled illumination source, combined with an ambient light-blocking feature, can significantly enhance light isolation and improve the signal-to-noise ratio^43^.

#### Location

Considerations for skin tone assessment depend in part on the location of the patient population under study.

For example, an outpatient clinic may be a single location where ambient lighting and temperature may be more easily standardized. Patients are often mobile, making it more feasible to incorporate skin tone measurements on less accessible sun-protected body parts (e.g., lower back). These may be difficult when collecting remote photos from a patient’s home where lighting may not be standardized and number of body parts for measurement may be limited. Additionally, longitudinal study design may need to account for patients’ variable sun exposure. Previous studies have included non-sun exposed body parts, advised participants to avoid sun exposure and/or require the use of sunscreen on a daily basis to attempt to address this^44^.

In contrast, an inpatient population presents complications when attempting to achieve a more fixed environment for data collection and synchronization of measurements. A more fixed environment for data collection can potentially be improved by understanding the unique workflow of standard care and adjusting each patient’s room to mimic a standardized environment. Although dependent on study design, study procedures may need to take into consideration patient health status, iatrogenic complications, patient mobility, and other external factors that could potentially hinder temporal aspects that are essential for the completion of measurements. For instance, in the context of pulse oximetry, short timepoints for data collection may be needed to minimize potential discrepancies between arterial blood gas (ABG)-pulse oximetry measurements and skin tone readings^45^.

#### Dataset balance—skin tone and race

Clinical research of all kinds must incorporate racial and ethnic diversity to ensure results are generalizable, especially when evaluating devices for clinical use^46,47^. Underrepresentation of minority groups may lead to a higher risk of adverse reactions or reduced efficacy. In fact, the NIH has an issued policy and guideline requiring all phase III clinical trials to ensure analysis by sex/gender, race, and/or ethnicity^48^. However, multiple studies have demonstrated large variations in skin tone within racial and ethnic subgroups^49^, and skin tone may directly influence bias of technologies beyond race. This highlights the potential need to balance datasets specifically by skin tone in addition to race. Ensuring dataset balance by skin tone may pose several challenges. Since skin tone varies within a person and across time, one may consider balancing only by constitutive body site or averaging skin tone from multiple body sites. Both skin tone and racial/ethnic dataset variation will enhance the generalizability of research results. However, investigators may consider balancing by either skin tone or race with a minimum threshold of other parameters based on their research question. Initial explorations by the Food and Drug Administration are soliciting input on methods to integrate skin tone measures into clinical device studies, but a standard has not been established^50^.

#### Considerations for administered visual scales

Visual scales are low-cost tools that can distinguish skin tones with relatively high reliability, require minimal training, are widely available, and can be utilized in various forms of analyses (retrospective, prospective, and post-hoc). Limitations of these scales are that they are influenced by user perception (e.g., color blindness) and environmental conditions (e.g., ambient lighting). There are also several visual scales available which can make comparison of skin tone data across studies difficult.

Few studies have assessed the relative utility of visual scales. FST was designed as a questionnaire to determine tanning and burning propensity, but when used as a proxy for skin tone is only weakly associated with a visual color scale (*p* < 0.0001)^51^. There have been attempts to create RGB-defined FST visual scales, although these have not been widely adopted^52^. The Von Luschan scale has been shown to be highly correlated with narrow band spectrophotometry with one study showing correlation of VLS and Melanin + erythema index to be 0.90 (*p* < 0.001)^53^. The scales with more levels (Pantone, Taylor Hyperpigmentation) offer greater granularity and shade range for skin tone assessment, but may be more challenging to reliably administer. For the Pantone scale, one study investigating vascularized allotransplantation matching found inter-rater skin tone assessment to be fair (*k* = 0.454) and intra-rater reliability to be substantial (*k* = 0.725)^54^. A newer, 10-point Monk scale shows high reliability for crowdsourced annotators (ICC 0.86–0.94), but has not been tested in a medical context^55^. Price may also be a consideration when choosing a scale. While the Monk scale is freely available and free to use and the FST questionnaire is easily accessible online, the Pantone scale is only available for purchase.

#### Considerations for color measurements tools

Colorimetric and spectrophotometric devices are used in a wide range of study designs to assess skin tone by quantifying melanin, erythema, and overall skin pigmentation. Common reflectance colorimetric and spectrophotometric devices are composed of an illuminator, standard observer, and a tristimulus measurement system. The illuminator of the instrument applies a fixed light source to a desired surface and specific wavelengths are then isolated to obtain color details without influence of outside lighting conditions when pressed gently to the skin. Colorimetric and spectrophotometric devices are easily-operated, non-invasive methods of measurement to achieve objective skin tone measurement. In varying clinical settings, handheld colorimetric and spectrophotometric devices (e.g., Delfin SkinColorCatch, Konica Minolta, and Variable Spectro) may be easier to use to assess body parts while also maintaining patient comfort. Although the devices demonstrate moderate to high interobserver reliability, devices are potentially high-cost, and most devices have not had large-scale published validation. A few studies have attempted to compare the utility of objective color measurement tools, but were limited in scope^33,56,57^. Consequently, a particular type of colorimetric/spectrophotometric device has not been proven to be superior^33^.

#### Considerations for cameras

Cameras are widely used, and available in the pocket of almost everyone a patient interacts with across the medical practice. There are also large datasets of images that have been acquired to train AI algorithms and other applications^11,58^. However, it can be challenging to extract skin tone information from a photograph alone. Camera type, export compression level, and lighting will be critical to consider^59^. One of the most important modifiable factors is the white balance, which affects the relationship between red, green, and blue pixel values^60^. The use of cross polarizing filters can help reduce specular reflection^61^ and improve skin tone evaluation, especially in darker toned individuals^43^. Reference color charts or color calibration cards (eg. X-Rite, Douglas, DSC Labs, QPcard, Macbeth) can be used within the frame of the image or before/after in identical conditions to improve reproducibility across devices, but will not be available for retrospectively captured images^59,60^. After acquisition, proper image processing is necessary to maintain color accuracy across mediums. Although popular image storage mediums like JPEG increase computational efficiency, image compression can lead to artifact and lost image parameter information. Therefore, RAW image format may be helpful for maintaining color consistency, although large file size may limit its utility, and many photography devices (eg. phones) cannot acquire in RAW format^32^.

#### Scale/device reliability

The process of evaluating device bias against skin tone measurement is nascent. The portability and low cost benefits of visual scales will need to be balanced against the potential increased accuracy of color measurement technologies that also include continuous measurement compared to categorical bins. Having at least two raters use visual scales and conducting triplicate readings for color measurement tools will increase color precision. When possible, comparison between multiple color measurement instruments will be valuable to the specific study and field.

## Discussion

Consideration of skin tone in device validation studies across medicine is important to reduce bias against patients with darker skin tones that exists in pulse oximetry, AI diagnosis, and many other areas of medicine. These biases will worsen existing healthcare disparities unless they are addressed and measured directly. In many cases, race and ethnicity play specific roles in equity focused-medicine and biased outcomes arise due to these socio-demographic factors^62^. A considerable amount of research is race and ethnicity focused, but for technologies that rely on light for measurement, their bias may be specifically related to skin tone. This highlights the need for increased awareness of limitations of current medical devices associated with systematic error and pronounced inaccuracies among patients with a darker skin tone.

In this review, we highlight common tools used for skin tone measurement and discuss pertinent study design considerations for accurate skin tone assessment. There is no current gold standard tool as each possesses relative pros and cons, and validation is largely absent. Visual scales are more readily available for prospective or post-hoc analysis, but may be influenced by user perception, while color measurement tools offer objective, sensitive measurements but can be expensive with variable reliability. In addition to tool choice, investigators should consider how patient level factors may affect skin tone validity, including selection of a body site, consideration for medical conditions affecting skin tone, and minimization of perfusion-related color changes. Furthermore, creation of a standardized environment with consistent lighting and camera settings will promote improved color consistency. This paper is a narrative review and therefore results are limited by the non-systematic approach. Further, the study is not powered to directly compare the utility of skin tone assessment modalities or quantify the potential effect of study design parameters on skin tone accuracy.

When prospectively evaluating devices that may be influenced by skin tone, incorporation of skin tone measurement will play an important role in considering these potential biases. The current review offers researchers a tool to aid in development of skin tone assessment protocols. We encourage researchers to continue to focus on validating devices against a diverse and representative dataset, and when possible, to make public the skin tone measurements for future use and calibration.

In conclusion, increasing evidence shows bias and increased error in noninvasive tools across medicine in patients with darker skin tones. We provide guidance and consideration when conducting skin tone assessments using administered scales (eg. Fitzpatrick, Pantone, Monk) and color measurement tools (colorimeters, spectrophotometers), encouraging device validation to include at least one color measurement tool. As our awareness as investigators consider skin tone as a variable in future work, we will be able to reduce skin tone biases in medical devices.

### Supplementary information

MSK - Protocol Checklist

Duke - Protocol Checklist

## Data Availability

All analyzed data is included in the published article.
