# Supplementary material for: A survey of skin tone assessment in prospective research
Source: NPJ Digit Med. 2024 Jul 17;7:191. doi: 10.1038/s41746-024-01176-8 (PMC11252344; doi:10.1038/s41746-024-01176-8)

MSK Skin Tone Protocol Checklist:

- ○ All assessments were performed in a single room with standardized environment (humidity/temperature) and consistent ambient lighting. Patients acclimated to the environment for 5 minutes before any color assessments.
- ○ Demographic information
  - Race (White, Black, Asian, American Indian/Alaskan, Hawaiian/Pacific, Other)
  - Ethnicity (Hispanic vs Non-Hispanic)
- ○ Fitzpatrick questionnaire
  - Tanning propensity (Do not tan, tan poorly, tan after burn, tan easily, tans easily darkly, always tan darkly)
  - Burning propensity (Always burn, easily burn, burn then tan, burn minimally, rarely burn, never burn)
  - Natural Hair Color
  - Natural Eye Color
- ○ Patient Self perceived Pantone and Monk at inner wrist
- ○ Up to 10 lesions and non-lesion anatomic site were marked with a sticker
  - Constitutive sites: inner upper arm, ventral forearm, abdomen, lower back
  - Facultative sites: forehead, dorsal forearm, chest, upper back, shin, calf, sole
- ○ Total Body Photography in white light and cross polarized light
- ○ Pantone perception by 2 raters, Monk perception by 2 raters
- ○ Pantone color card iPhone App at inner wrist (x2), outer forearm, inner upper arm
- ○ Dermoscopic images with camera #1 of each lesion & anatomic site in non-contact white and cross polarized and contact white and cross polarized
- ○ Dermoscopic images with camera #2 at lesion #1, outer forearm, inner wrist
- ○ Colorimeter readings at inner wrist (x3) forearm (x3), upper inner arm(x3), skin around lesion 1 (x3)

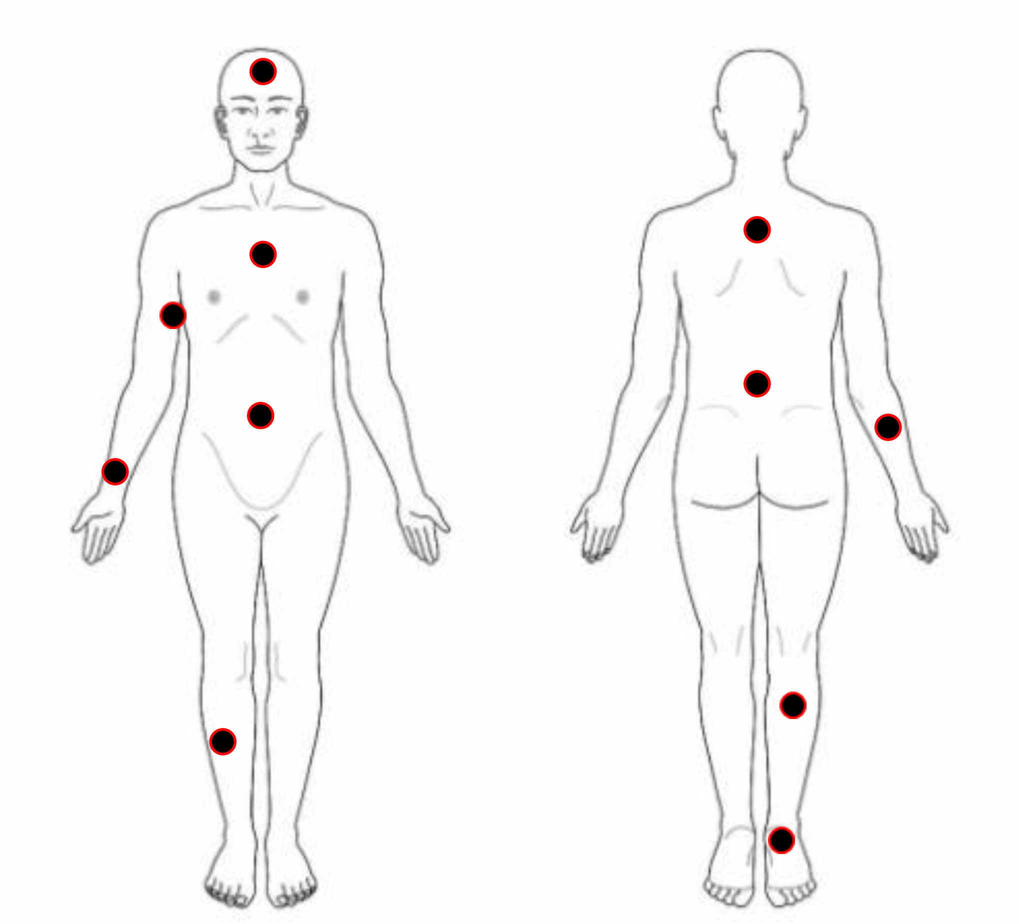

Supplement: Supplementary file 1 — MSK - Protocol Checklist [file 41746_2024_1176_MOESM1_ESM.pdf]
