# Supplementary material for: A survey of skin tone assessment in prospective research
Source: NPJ Digit Med. 2024 Jul 17;7:191. doi: 10.1038/s41746-024-01176-8 (PMC11252344; doi:10.1038/s41746-024-01176-8)

**Duke Pulse Oximetry and Skin Tone Protocol Checklist:**

- All assessments were performed on patients admitted to Duke University Hospital.
- Patients were eligible if they consented within 7 days of ABG-pulse oximetry pairs. Study environments were standardized for lighting conditions.
- Exclusion criteria:
    - < 18 years of age
    - Large areas of skin discoloration due to current rash, vitiligo, jaundice, and wounds/bruising at measurement sites
    - Vascular complications (including known peripheral arterial disease)
    - Inability to remove nail polish
- Demographic information:
    - Race (White, Black/African American, Asian, American Indian/Alaskan Native, Native Hawaiian/Other Pacific Islander, More than one race, Other)
    - Ethnicity (Non-Hispanic vs Hispanic)
- Clinical team and study team confirmed pulse oximeter location and brand as well as if patient is under a standard care temperature protocol.
- Additional clinical data, such as vitals and laboratory results, were also extracted from the EHR.
- Skin tone assessments were taken on 16 locations of the body. The body parts assessed are identified by a black dot below. Ventral and dorsal measurements were collected from the earlobe and the left and right fingers, toes, and palms. Additional measurements were taken over the forehead and sternum.
- Assessment tools consisted of:
    - Temperature devices (General-range and Clinical-range)
    - Administered visual scales (Fitzpatrick Skin Type, Monk Skin Tone, and Von Luschan)
    - Colorimetric/spectrophotometric devices
        - Konica Minolta CM700D
        - Delfin SkinColorCatch
        - Spectro Variable
    - Digital photography (Google Pixel 4a and iPhone SE 2020)

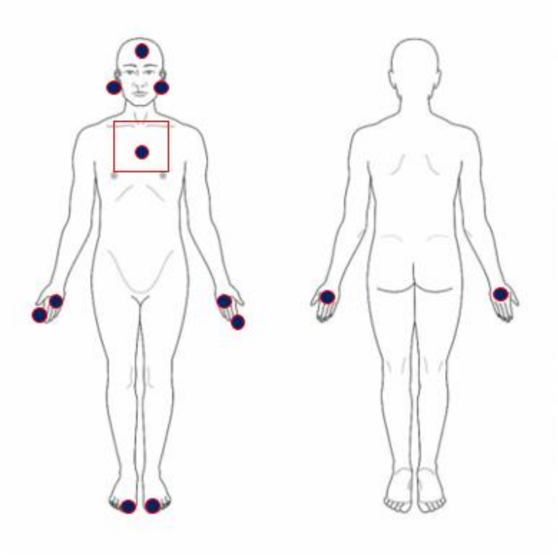

Supplement: Supplementary file 2 — Duke - Protocol Checklist [file 41746_2024_1176_MOESM2_ESM.pdf]
